# Brain Gαi*_2_*-subunit proteins and the prevention of salt sensitive hypertension

**DOI:** 10.3389/fphys.2015.00233

**Published:** 2015-08-19

**Authors:** Casey Y. Carmichael, Richard D. Wainford

**Affiliations:** The Department of Pharmacology and Experimental Therapeutics, The Whitaker Cardiovascular Institute, Boston University School of MedicineBoston, MA, USA

**Keywords:** central G**α**i_2_ proteins, blood pressure regulation, central G-protein coupled receptors, renal sympathetic nerves, sympathetic nervous system, salt-sensitive hypertension, sodium homeostasis

## Abstract

To counter the development of salt-sensitive hypertension, multiple brain G-protein-coupled receptor (GPCR) systems are activated to facilitate sympathoinhibition, sodium homeostasis, and normotension. Currently there is a paucity of knowledge regarding the role of down-stream GPCR-activated Gα-subunit proteins in these critically important physiological regulatory responses required for long-term blood pressure regulation. We have determined that brain Gαi_2_-proteins mediate natriuretic and sympathoinhibitory responses produced by acute pharmacological (exogenous central nociceptin/orphanin FQ receptor (NOP) and α_2_-adrenoceptor activation) and physiological challenges to sodium homeostasis (intravenous volume expansion and 1 M sodium load) in conscious Sprague–Dawley rats. We have demonstrated that in salt-resistant rat phenotypes, high dietary salt intake evokes site-specific up-regulation of hypothalamic paraventricular nucleus (PVN) Gαi_2_-proteins. Further, we established that PVN Gαi_2_ protein up-regulation prevents the development of renal nerve-dependent sympathetically mediated salt-sensitive hypertension in Sprague–Dawley and Dahl salt-resistant rats. Additionally, failure to up-regulate PVN Gαi_2_ proteins during high salt-intake contributes to the pathophysiology of Dahl salt-sensitive (DSS) hypertension. Collectively, our data demonstrate that brain, and likely PVN specific, Gαi_2_ protein pathways represent a central molecular pathway mediating sympathoinhibitory renal-nerve dependent responses evoked to maintain sodium homeostasis and a salt-resistant phenotype. Further, impairment of this endogenous “anti-hypertensive” mechanism contributes to the pathophysiology of salt-sensitive hypertension.

## Selectivity of central Gα proteins

**Figure 1:**
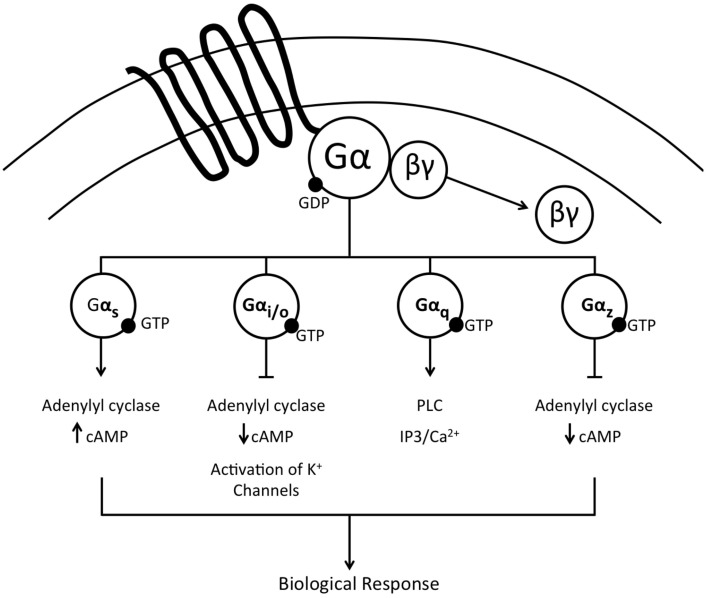
**Schematic representation of the major Gα protein signal transduction pathways activated following ligand binding at a G-protein coupled receptor**.

G-proteins are a family of heterotrimeric proteins composed of α, β, and γ subunits. Following ligand binding at a transmembrane G-protein coupled receptor (GPCR), signal transduction is initiated by α-subunit mediated exchange of GDP for GTP and the dissociation into activated α and βγ complexes that initiate downstream signal transduction to ultimately evoke a biological response. This review is focused on delineating the central actions of Gα proteins in cardiovascular and fluid and electrolyte homeostasis. There are four major subtypes of Gα proteins, Gαi/o, Gαs, Gαz, and Gαq (Figure 1), discussed in this review, with a subsequent focus on Gαi_2_ proteins. The Gαi/o and Gαz classes of Gα proteins principally inhibit adenylate cyclase, thereby resulting in reduced cAMP levels. Additionally, via associated βγ subunits Gαi/o proteins also activate potassium channels. In contrast, Gαs proteins stimulate the activity of adenylate cyclase to drive increased cAMP production. The final subtype investigated in these studies, Gαq, does not impact cAMP levels, and instead activates phospholipase C (PLC), resulting in increased levels of intracellular inositol triphosphate (IP3) and modulation of calcium release. It has been demonstrated in cell culture systems that selective recruitment and/or availability of Gα-subunit proteins plays a critical role in determining the intracellular signaling responses to GPCR activation following ligand binding (Nasman et al., 2001). The specific intracellular actions evoked by selective action of individual Gα proteins has been elegantly elucidated, in part, via studies conducted on the nociceptin/orphanin FQ receptor (NOP). Activation of the NOP GPCR results in signal transduction via Gαi_1−3_, Gαo, Gαz, and Gαq proteins to initiate multiple cellular responses, including activation of potassium channels and inhibition of calcium channels, adenylyl cyclase, and MAPK phosphorylation—all of which modulate neurotransmitter release and neuronal activity (Reinscheid et al., 1995; Chan et al., 1998; Hawes et al., 1998; Jeong and Ikeda, 1998; Yung et al., 1999; Tso and Wong, 2006). *In vitro* studies on the α_2_-adrenoceptor, an extensively studied key GPCR in cardiovascular regulation, have demonstrated signal transduction via Gαi_1−3_, Gαo, Gαs, Gαz, and Gαq subunit proteins which principally modulate adenylate cyclase activity (Remaury et al., 1993; Nasman et al., 2001; Hein, 2006). However, despite the elucidation of signal transduction pathways *in vitro* for the NOP and α_2_-adrenoceptor (among others), there are essentially no data at present correlating intracellular Gα protein pathways to the physiological responses elicited by central GPCR activation *in vivo*.

## Brain Gα proteins and pharmacological GPCR activation

To investigate the potential of selective physiological actions of central Gα-proteins following ligand binding at a GPCR *in vivo*, we elected to use the highly selective GPCR agonist nociceptin/orphanin FQ (N/OFQ) (Reinscheid et al., 1995; Calo et al., 2005; Krowicki and Kapusta, 2006). N/OFQ, an opioid-like peptide, produces highly reproducible cardiovascular depressor and diuretic responses when administered centrally to conscious rats via actions on the NOP receptor (Kapusta et al., 1997, 1999; Kapusta and Kenigs, 1999; Kakiya et al., 2000). Prior *in vitro* studies suggested that central NOP receptors signal through Gαi_1_, Gαi_2_, Gαi_3_ (Hawes et al., 1998), Gαo_A, B_ (Tso and Wong, 2006), Gαz (Chan et al., 1998; Jeong and Ikeda, 1998), and Gαq (Yung et al., 1999) transduction pathways. We therefore examined how inhibition of central Gαi/Gαo proteins via pertussis toxin (Katada and Ui, 1982; Gullapalli and Ramarao, 2002) or targeted selective down-regulation of central Gαz or Gαq proteins via intracerebroventricular (i.c.v.) oligodeoxynucleotide (ODN) pre-treatment (Rossi et al., 1995; Standifer et al., 1996; Silva et al., 2000; Hadjimarkou et al., 2002) impacted the characteristic cardiovascular, renal excretory, and plasma vasopressin (AVP) responses to i.c.v. injection of N/OFQ in conscious Sprague–Dawley rats. In control saline vehicle or scrambled ODN pre-treatment groups, i.c.v. administration of N/OFQ evoked bradycardia, hypotension, and diuresis. The hypotension and bradycardia, but not diuresis, to N/OFQ was abolished in pertussis toxin-pretreated rats in which the actions of brain Gαi/Gαo proteins were inhibited. In contrast, i.c.v. ODN pretreatment, which significantly and specifically down-regulated central Gαz or Gαq protein expression as previously reported (Rossi et al., 1995; Standifer et al., 1996; Silva et al., 2000; Hadjimarkou et al., 2002) had a significant effect on the diuresis to central N/OFQ without impacting the cardiovascular responses. Owing to the known role of N/OFQ in suppressing AVP release, reducing water reabsorption in the collecting ducts of the kidneys and promoting diuresis, we determined that the effects of central Gαz or Gαq down-regulation on N/OFQ diuresis were mediated by blunting (Gαz) or augmenting (Gαq) N/OFQ evoked suppression of AVP release (Wainford et al., 2008). These studies demonstrate that in conscious Sprague–Dawley rats the GPCR ligand N/OFQ acts centrally via the NOP receptor to activate individual Gα protein signaling pathways that control cardiovascular (Gαi/Gαo) vs. renal excretory (Gαz or Gαq) function.

Extending our finding that central Gαz/Gαq protein pathways play a pivotal role in modulating (inhibiting vs. stimulating, respectively) AVP release and thereby diuresis to central N/OFQ (Wainford et al., 2008), we examined the role of brain Gαz/Gαq proteins in the regulation of AVP during a physiological challenge. During high salt intake, Dahl salt-sensitive (DSS) rats exhibit elevated plasma AVP (Bayorh et al., 1998; Gunnet et al., 2008). In contrast, in salt-resistant rat phenotypes [i.e., Sprague–Dawley (SD) and Dahl salt-resistant (DSR) rats], increased dietary salt intake does not alter circulating AVP levels (Serino et al., 2001; Wainford and Kapusta, 2009). A key finding of these investigations is that in response to a high salt diet, SD and DSR rats down-regulate Gαq protein levels within the hypothalamic PVN, a major central site involved in AVP synthesis and release (Landgraf et al., 1990; Nielsen et al., 1993; Grindstaff and Cunningham, 2001). This site-specific down-regulation of PVN Gαq proteins was correlated with the ability of SD and DSR rats to maintain unchanged plasma AVP levels and fluid/electrolyte balance in the face of a high salt challenge (Wainford and Kapusta, 2010). In contrast, DSS rats do not exhibit such site-specific down-regulation of Gαq proteins, which may contribute to excess AVP release in this rat phenotype. Owing to the impact of PVN Gαq proteins on the regulation of AVP release, we speculate that within the PVN, the changes in Gαq protein expression are predominantly localized to the magnocellular neurons. Of physiological relevance, ODN-mediated down-regulation of brain Gαq proteins restored plasma AVP in salt-challenged DSS rats to levels observed prior to consuming a high salt diet (Wainford and Kapusta, 2010). Moreover, diuresis stimulated by pharmacological or physiological manipulations that are known to evoke this response exclusively or in part via suppression of AVP release (Kondo et al., 1993; Kakiya et al., 2000; Krowicki and Kapusta, 2006; Ruginsk et al., 2007) was completely or partially restored (Wainford and Kapusta, 2010). Therefore, the down-regulation of PVN Gαq proteins plays a critical counter-regulatory role in preventing AVP hypersecretion by reducing the ability of endogenous GPCR ligands to trigger AVP release, and may represent a new therapeutic target in pathophysiological states featuring AVP dysregulation (e.g., heart failure, salt-sensitive hypertension). However, the potential impact of PVN Gαz/Gαq proteins in other pathophysiological states featuring AVP excess remains to be determined and is not the focus of the remainder of this review.

Our prior studies also established a role for brain Gαi/Gαo proteins to mediate hypotension and bradycardia in response to central activation of the NOP receptor (Wainford et al., 2008), but did not determine which specific Gαi/Gαo protein is responsible for the observed effects. To address this issue and provide clarity as to the individual roles of brain Gαi/Gαo proteins in cardiovascular regulation, conscious Sprague–Dawley rats were administered gaunabenz, a selective α_2_-adrenoceptor agonist (Degoute, 2007). The α_2_-adrenoceptor is intimately involved in the central control of cardiovascular function and fluid and electrolyte homeostasis (Ruffolo et al., 1991; Nasman et al., 2001). *In vitro* studies have demonstrated that immediately following post-ligand binding, α_2_-adrenoceptors activate downstream Gαi(1–3), Gαo, and Gαs protein signal transduction pathways (Remaury et al., 1993; Eason and Liggett, 1995). Ligand-binding at central α_2_-adrenoceptors in conscious rats evokes bradycardia, hypotension, sympathoinhibition (Grisk and Dibona, 1998; Huang and Leenen, 1998), diuresis and natriuresis (Gellai and Edwards, 1988; Menegaz et al., 2001). Using a highly targeted ODN approach to selectivity down-regulate central Gαi_1_, Gαi_2_, Gαi_3_, Gαo, or Gαs proteins (Rossi et al., 1995; Standifer et al., 1996; Silva et al., 2000; Hadjimarkou et al., 2002), we determined that the hypotensive and natriuretic responses to selective central α_2_-adrenoceptor activation are abolished by Gαi_2_ down-regulation, or are converted to a pressor response by Gαs down-regulation. We speculate these responses reflect altered sympathetic outflow to multiple organ systems (e.g., renal, splanchnic). In contrast, the profound α_2_-agonist-stimulated bradycardia and diuresis was unaltered by prior down-regulation of individual brain Gαi(1–3), Gαs, or Gαo proteins (Wainford and Kapusta, 2012). These data suggest potential redundancy of Gα protein pathways to regulate heart rate. In light of our prior findings about AVP regulation (described above), we speculate the diuretic response to central α_2_-adrenoceptor activation is regulated by Gαz/Gαq due to a significant component of this response being mediated by suppression of AVP release (Brooks et al., 1986; Cabral et al., 1998). Together, these studies provide new insight into the intracellular mechanism of action of brain α_2_-adrenoceptors *in vivo* and document that the physiological responses evoked by receptor activation are greatly influenced by the availability and/or brain protein expression levels of individual downstream Gα proteins (Wainford and Kapusta, 2012). Further, these data provide compelling *in vivo* support for the pharmacological paradigm of functional selectivity (Patel et al., 2010) as predicted from *in vitro* studies and mathematical models (Nasman et al., 2001; Hein, 2006).

## Brain Gαi_2_ proteins and acute physiological GPCR activation

**Figure 2:**
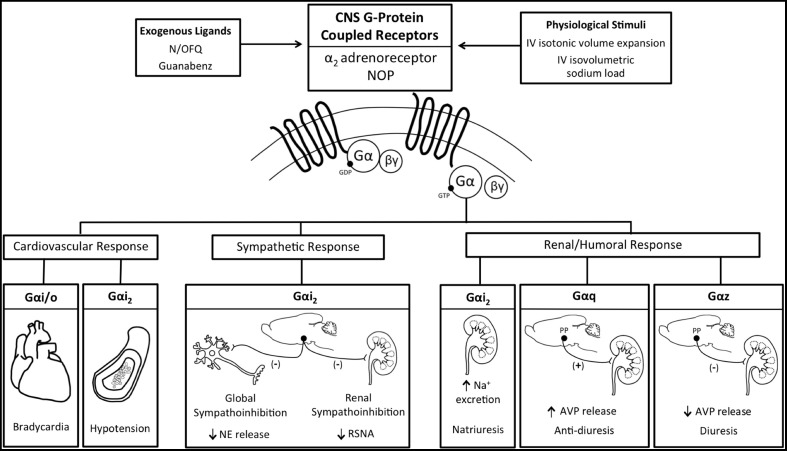
**Schematic representation of the functional selectivity of G-protein coupled receptor activated Gα protein signal transduction pathways in mediating the physiological responses evoked by central exogenous administration of N/OFQ and Guanbenz or the physiological stimuli of an iv volume expansion or sodium load**. AVP, vasopressin; NE, Norepinephrine; NOP, nociceptin/orphanin FQ receptor; RSNA, renal sympathetic nerve activity.

Maintenance of fluid and electrolyte homeostasis in response to acute increases in sodium intake occurs via complex integrated neural, humoral, and hemodynamic pathways to regulate the renal excretion of sodium. If these endogenous pathways are impaired, salt-sensitive hypertension can develop. A key component of the renal handling of sodium and water, and thus blood pressure, in response to alterations in plasma sodium, is the regulation of sympathetic outflow and renal sympathetic nerve traffic (Guyton, 1991; Lohmeier et al., 1999; DiBona, 2004, 2005; May et al., 2009), which is directly influenced by multiple brain GPCR systems (e.g., the α_2_-adrenoreceptor) (Ruffolo et al., 1991; Nasman et al., 2001). However, the central mechanisms regulating sympathetic outflow (global and renal specific) to facilitate natriuresis during elevations in plasma sodium content remain unclear (DiBona, 2005; Kompanowska-Jezierska et al., 2008; Bie, 2009). Consequently, we elected to examine the role of endogenous brain Gαi_2_ protein-gated signal transduction pathways in mediating the sympathetic and natriuretic responses to acute physiological challenges [i.v. volume expansion (VE) and i.v. sodium load] that impact the renal handling of sodium, in part, by suppressing central sympathetic outflow (Haselton et al., 1994; Kapusta and Obih, 1995; DiBona and Kopp, 1997; Singer et al., 1998; Manunta et al., 2011). Selective ODN-mediated down-regulation of brain Gαi_2_ proteins (1) abolished the renal sympathoinhibitory response and attenuated the natriuresis to VE (Kapusta et al., 2012) and (2) abolished the global sympathoinhibitory response and attenuated the natriuresis to a 1 M sodium load (Wainford et al., 2013) via a mechanism requiring intact renal sympathetic nerves. Demonstrating the central nature of this effect, it is notable that kidney function was maintained following down-regulation of brain Gαi_2_ proteins, as demonstrated by the natriuretic and diuretic responses to i.v. bolus administration of furosemide in Gαi_2_ ODN-treated rats (Kapusta et al., 2012). The results generated from these physiological studies demonstrate that central Gαi_2_ proteins are involved in mediating the renal excretory responses to these acute challenges to sodium homeostasis via an inhibitory influence on sympathetic outflow, specifically renal sympathetic nerve activity. This conclusion is supported by evidence that down-regulation of brain Gαi_2_ proteins prevented the suppression of global sympathetic outflow [assessed by circulating norepinephrine (NE) levels] during a sodium load (Wainford et al., 2013). Moreover, in rats implanted with a renal nerve recording electrode, brain Gαi_2_ protein down-regulation abolished isotonic saline volume expansion (5% body weight) evoked suppression of renal sympathetic nerve activity (Kapusta et al., 2012). Bilateral renal denervation to remove the influence of the renal sympathetic nerves on kidney function prevented Gαi_2_ protein down-regulation from altering the natriuretic response to either the i.v. VE or 1 M sodium load (Kapusta et al., 2012; Wainford et al., 2013). Linking our data generated following acute exogenous central GPCR activation (described above) to these physiological studies provides evidence that central α_2_-adrenoceptors are the principal brain GPCR involved in producing renal sympathoinhibition, and consequently natriuresis, in response to acute VE (Patel, 1991). Therefore, we postulate that endogenous α_2_-adrenoceptor mediated activation of brain Gαi_2_ protein-gated pathways represents a key mechanism by which sympathoinhibition and natriuresis occurs *in vivo* in response to acute challenges to sodium homeostasis. Collectively, these data report a central molecular mechanism that plays a key role in modulating the level of central sympathetic outflow, blood pressure, and natriuresis in response to acute pharmacological or physiological stimuli (Figure 2). Further, these data establish that central Gαi_2_ proteins impact sympathetic outflow predominantly via regulation of the renal sympathetic nerves. The present studies extend our knowledge of the CNS regulatory mechanisms influencing the kidney to maintain sodium homeostasis. Given the intimate association between fluid and electrolyte homeostasis and the long-term control of arterial pressure, we speculate that Gαi_2_-protein mediated suppression of central sympathetic outflow may represent a new component in integrated autonomic regulatory processes that govern the long-term regulation of systemic arterial blood pressure. The importance of enhancing our understanding of the underlying cellular signaling pathways involved in the neural control of sodium excretion in health and disease is highlighted by the multiple pathophysiological disease states that exhibit sodium retention, including heart failure and certain models of hypertension, particularly salt-sensitive hypertension (Bayorh et al., 1998; Lastra et al., 2010).

## Brain Gαi_2_ proteins and salt-resistance

Due to the dramatic impact of hypertension on human health and the prevalence of salt-sensitive hypertension in approximately 50% of hypertensive patients (Meneton et al., 2005; Franco and Oparil, 2006; Whelton et al., 2012; Kotchen et al., 2013), we elected to study the potential role of central Gαi_2_ proteins in the pathophysiology of salt-sensitive hypertension. It is well established in normotensive salt-resistant subjects that neural (renal sympathetic) and humoral (angiotensin-aldosterone) sodium-retaining mechanisms are suppressed to counter the influence of dietary salt-intake on central sympathetic outflow and systemic cardiovascular hemodynamics (Lohmeier et al., 1999; Brooks et al., 2005; Osborn et al., 2008). Consequently, we examined the role of central Gαi_2_ proteins in facilitating sodium homeostasis during a 7-day dietary sodium restriction or supplementation in the Sprague–Dawley rat. In control scrambled ODN-treated rats, 7-days of altered dietary sodium intake did not alter blood pressure and evoked a site-specific PVN decrease (sodium deficiency) or increase (sodium excess) in PVN Gαi_2_ proteins (Kapusta et al., 2012). The sodium-stimulated changes in Gαi_2_ protein expression were restricted to the PVN and did not occur in other hypothalamic regions (e.g., SON, posterior hypothalamus). Further, dietary sodium restriction or excess did not alter expression levels of Gαi_1_, Gαi_3_, or Gα_o_ proteins in any brain site examined. The observation of sodium-evoked site and subunit-specific changes in PVN Gαi_2_ protein expression is of considerable pathophysiological interest as this brain site plays a key role in responding to alterations in body fluid sodium concentration/osmolality (Toney et al., 2003) and the central control of sympathetic outflow, sodium homeostasis, and blood pressure (Leenen et al., 2002; Kenney et al., 2003; He et al., 2005; Carmichael and Wainford, 2015). In rats adapted to a high-sodium diet (0.9% NaCl drinking water), acute ODN-mediated down-regulation of central Gαi_2_ proteins resulted in the development of sodium retention, global sympathoexcitation (i.e., increased circulating NE), and moderately elevated blood pressure. These findings provide further support for the long established Guytonion hypothesis of the intimate connection between fluid and electrolyte homeostasis and the long-term regulation of blood pressure (Guyton, 1991). Additionally, in accordance with recent hypotheses (Rodriguez-Iturbe and Vaziri, 2007; Fujita and Fujita, 2013; Stocker et al., 2013), our data indicate that enhanced central sympathetic outflow plays a role in these regulatory processes. The PVN as a potential locus of Gαi_2_protein mediated regulation of sympathetic outflow is not unexpected, due to the critical role of the PVN in mediating sympathoinhibition (Coote, 1995; Akine et al., 2003; Frithiof et al., 2009), particularly in response to increased plasma and/or cerebrospinal fluid sodium in salt-resistant subjects (Brooks et al., 2005; DiBona, 2005; He et al., 2005). These data demonstrate that PVN Gαi_2_ protein pathways play a role in maintaining fluid and electrolyte balance during alterations in sodium intake by controlling the influence of the sympathetic nervous system on natriuresis and suggest a role for these proteins in long-term blood pressure regulation (Kapusta et al., 2012).

**Figure 3:**
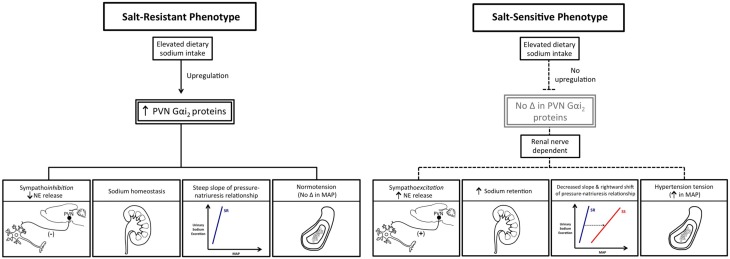
**Schematic representation of the differential impact of elevated dietary sodium intake on PVN Gαi_2_ protein expression and the consequences of this on central sympathetic outflow, sodium homeostasis, and blood pressure in salt-resistant vs. salt-sensitive phenotypes**. MAP, mean arterial pressure; NE, Norepinephrine; PVN, paraventricular nucleus.

Currently, the central molecular mechanisms that mediate sympathoinhibition in response to excess dietary sodium intake as found in the typical western diet [Center for Disease Control and Prevention (CDC), 2010] remain to be fully elucidated. Based on our prior observations, we hypothesized that in salt-resistant phenotypes (e.g., Sprague–Dawley rat) subjected to a chronic elevation in dietary sodium intake, brain Gαi_2_ protein signaling pathways will be augmented to maximize inhibition of central sympathetic outflow to the kidneys via the renal sympathetic nerves and thereby facilitate sodium excretion. Further, we predict that blockade of this central Gαi_2_ protein sympathoinhibitory pathway will trigger sodium and water retention and the development of salt-sensitive hypertension. To chronically down-regulate central Gαi_2_ proteins, our acute ODN administration protocol was adapted to utilize an alzet osmotic minipump connected to an indwelling i.c.v. cannula to deliver a continuous infusion of a control scrambled or Gαi_2_ ODN (Kapusta et al., 2013). During ODN infusion, naïve or previously bilaterally renal denervated male Sprague–Dawley rats were randomly assigned to receive a normal salt (0.4%) or high-salt (8.0%) diet for 21-days. In control scrambled ODN-infused rats, salt-loading which did not alter blood pressure, evoked a site-specific increase in hypothalamic paraventricular nucleus (PVN) Gαi_2_ protein levels and suppression of circulating norepinephrine content and plasma renin activity. To demonstrate the functional significance of sodium-evoked PVN specific increase in Gαi_2_ proteins, we examined how chronic ODN-mediated down-regulation of brain Gαi_2_ protein levels impacted blood pressure and neural/humoral sodium retaining mechanisms during high salt intake. In these studies, salt-loaded rats continuously infused i.c.v. with a Gαi_2_ ODN developed salt-sensitive hypertension (Kapusta et al., 2013). The development of salt-sensitive hypertension, featuring an impaired pressure-natriuresis response, during CNS Gαi_2_ protein down-regulation involves global sympathoexcitation that we postulate contributes to increased systemic arterial pressure over time through (1) increased vascular contractility and (2) enhanced renal tubular sodium reabsorption (DiBona and Kopp, 1997; Lohmeier et al., 1999). In contrast to an inhibitory influence on sympathetic activity and NE release, Gαi_2_ protein pathways do not impact the suppression of the renin-angiotensin system (as assessed by reduced plasma renin activity) during chronic increases in dietary sodium intake (Kapusta et al., 2013). In separate studies using the technique of chronic bilateral renal denervation, which remove the influence of the renal sympathetic nerves on kidney function, we were able to prevent the development of central Gαi_2_ ODN-induced salt-sensitive hypertension. In renal denervated animals, we did not observe global sympathoexcitation or resetting of the chronic pressure-natriuresis curve in response to elevated in dietary sodium intake (Wainford et al., 2013). These data demonstrate the physiological importance of PVN Gαi_2_ proteins as a sodium-activated “anti-hypertensive” central pathway which regulates the renal handling of sodium via the renal sympathetic nerves to prevent the development of salt-sensitive hypertension (Figure 3). Our findings support the inclusion of a role for enhanced central sympathetic outflow in the current modeling of the pathophysiology of hypertension that is based on the classical Guytonian theory of pressure natriuresis (Guyton, 1991). Further, our data highlight central Gαi_2_ proteins as a candidate mechanism that facilitates the communication between the central nervous system and the kidneys to maintain long-term blood pressure regulation.

## Brain Gαi_2_ proteins and salt-sensitivity

Among multiple proposed mechanisms, impaired sympathetic nervous system activity has been hypothesized to play a critical role in the development of salt-sensitive hypertension (Brooks et al., 2005; Osborn et al., 2009; Fujita and Fujita, 2013), a theory gaining traction following reports of increased sympathetic nervous system activity in animal models of salt-sensitive hypertension (Kandlikar and Fink, 2011; Foss et al., 2013) and salt-sensitive humans (Yatabe et al., 2010). Further, increasing attention is focusing on the role of the renal sympathetic nerves in blood pressure regulation following reports that renal nerve ablation attenuates hypertension in human subjects (Esler et al., 2012; Krum et al., 2014). Despite these recent advances, it is widely acknowledged that the central nervous system mechanisms that modulate central sympathetic outflow to the kidney to facilitate sodium homeostasis and blood pressure regulation require further investigation (Brooks et al., 2005; Ellison and Brooks, 2011; Stocker et al., 2013). Based on our prior findings, generated solely in the Sprague–Dawley rat phenotype, we hypothesized that increased dietary sodium intake upregulates PVN Gαi_2_ proteins to suppress central sympathetic outflow, particularly to the kidneys, to maintain fluid and electrolyte balance and normotension in multiple salt-resistant phenotypes. Extending this hypothesis, we predicted that failure to up-regulate PVN Gαi_2_ proteins in response to increased salt intake contributes to the pathophysiology of salt-sensitive hypertension.

Utilizing the Dahl rat phenotypes as an established model of salt-resistance vs. salt-sensitivity, we observed that high dietary sodium intake evoked a site-specific up-regulation of PVN Gαi_2_ protein levels in the DSR and Brown Norway rat phenotypes (Wainford et al., 2015). These findings extend our prior studies in Sprague–Dawley rats (Kapusta et al., 2012, 2013) and demonstrate that PVN Gαi_2_ protein up-regulation in response to increased salt-intake occurs in multiple rat phenotypes that exhibit salt-resistance. In contrast, in the DSS rat, which developed salt-sensitive hypertension, there was a failure to up-regulate PVN Gαi_2_ proteins (Wainford et al., 2015). This difference would suggest a molecular mechanism that contributes to DSS rat hypertension. During high salt-intake, central ODN-mediated Gαi_2_ protein down-regulation evoked sympathetically mediated salt-sensitive hypertension in the DSR rat and exacerbated the magnitude of salt-sensitive hypertension observed in the DSS phenotype. As determined via radiotelemetry, down-regulation of CNS Gαi_2_proteins evoked an elevation in blood pressure in the DSR rat upon high-salt challenge of approximately 20 mmHg in a 3-day period, followed by a prolonged and persistent increase in blood pressure over time. In the DSS rat, CNS Gαi_2_protein down-regulation also evoked an enhanced pressor response to salt-intake, after which the rate of development of hypertension was comparable between scrambled and Gαi_2_ treated animals. This rapid elevation in blood pressure following salt-intake and brain Gαi_2_protein down-regulation reflects a reduction in the slope of the blood pressure-sodium excretion relationship, indicative of increased short-term salt-sensitivity of blood pressure (Wainford et al., 2015). This would reset the pressure-natriuresis set-point to a higher level within several days, as observed in the current studies, and as hypothesized by current models of the development of salt-sensitive hypertension (Van Vliet et al., 2006; McLoone et al., 2009; Fujita and Fujita, 2013).

Chronic removal of the influence of the renal sympathetic nerves abolished central Gαi_2_ ODN-induced sympathetically mediated salt-sensitive hypertension in DSR rats and attenuated the development of salt-sensitive hypertension in DSS rats. These data support a direct role of renal sympathetic innervation of the kidney in the development of salt-sensitive hypertension (Wainford et al., 2015). Based on our prior studies, we speculate in intact animals in which the influence of brain Gαi_2_ was removed, there was a failure to suppress sympathetic outflow to the kidneys. This would have the consequence of increasing both renal NE release and NE-mediated sodium reabsorption leading to the observed sodium retention to drive the pathophysiology of salt-sensitivity. To confirm that impaired up-regulation of central Gαi_2_ proteins impacts salt-sensitivity in the DSS rat, we conducted studies using an 8-congenic DSS rat, which has chromosome 8 encoding the GNAI2 gene from the Brown Norway rat (Mattson et al., 2008). During high salt-intake, the 8-congenic DSS rat exhibited up-regulation of PVN Gαi_2_ proteins as well as attenuation of sodium evoked hypertension, sodium retention and sympathoexcitation (Wainford et al., 2015). As anticipated, because of the multifactorial nature of DSS hypertension, restoration of sodium-stimulated PVN Gαi_2_ protein up-regulation attenuated, but did not abolish, the hypertension. Collectively, these studies report that sodium evoked up-regulation of PVN Gαi_2_ proteins represents a conserved central molecular mechanism that is required to suppress renal sympathetic outflow to maintain sodium homeostasis and normotension in multiple salt-resistant rat phenotypes. Additionally, studies conducted in the DSS and 8-congenic DSS rat provide the first direct evidence that impairment of PVN Gαi_2_ signal transduction pathways contributes to the pathophysiology of salt-sensitive hypertension (Figure 3).

## Future directions and significance

Despite the insight our recent studies have provided over the last several years into the impact of central Gαi_2_proteins on the salt-sensitivity of blood pressure, several questions remain to be addressed. A critical issue remains the time course of up-regulation of hypothalamic PVN Gαi_2_ proteins in response to increases in dietary sodium intake. At present, all data generated by our laboratory represents end-point assessment after 21-days of increased dietary intake (Kapusta et al., 2013; Wainford et al., 2015). Our physiological data suggests up-regulation of Gαi_2_ proteins is an early event but this is yet to be confirmed. Further, identification of the mechanism driving the increased expression of hypothalamic PVN Gαi_2_ proteins in response to altered dietary sodium intake remains to be established. Despite our failure to detect changes in either plasma sodium or osmolality in SD, DSR, or Brown Norway rats during increased sodium intake in end-point measurements made on day-21 of high salt-intake (Kapusta et al., 2013; Wainford et al., 2015), we speculate minute changes in plasma sodium/osmolality following sodium ingestion (i.e., after a meal) are sensed by osmoreceptor/sodium-sensitive receptors located in the hypothalamic PVN, the circumventricular organs (Brooks et al., 2005; Stocker et al., 2013) or the renal afferent nerve terminals (DiBona and Kopp, 1997) and trigger the observed alterations in protein expression. An alternative potential mechanism driving the increase in PVN Gαi_2_ protein expression during salt intake may be sodium-evoked alterations in PVN neurotransmitter release (e.g., altered intra-PVN levels of glutamate and/or GABA) and that the complex inhibitory and excitatory actions evoked by these neurotransmitters may evoke altered PVN Gαi_2_ levels. The current studies strongly suggest the PVN as the locus of the observed actions of central Gαi_2_ proteins, however future microinjection studies, designed to down-regulate Gαi_2_ proteins site-specifically within the PVN are required to definitively establish the PVN as a brain site in which Gαi_2_ protein pathways act to produce sympathoinhibitory influences on the renal handling of sodium and water to counter the development of salt-sensitive hypertension. We speculate, given the profound impact of alterations in PVN Gαi_2_ on sympathetic outflow, that the observed changes in Gαi_2_ expression in response to increased salt intake occur in the parvocellular sympathetic-regulatory neurons, not the magnocellular neurons. An additional issue not addressed by our current work is the cellular mechanism(s) by which central Gαi_2_ proteins modulate sympathetic outflow, sodium homeostasis, and blood pressure. We postulate that central Gαi_2_ proteins influence these physiological parameters through alteration of neuronal firing patterns, via actions to inhibit adenylate cyclase activity and/or Ca^2+^ channels in sympathetic neurons, to evoke changes in the activity of the CNS to impact central sympathetic outflow. However, electrophysiological recordings conducted in conscious animals and in brain slice preparation studies are required to establish the underlying cellular mechanisms involved in these responses.

Our observation of an increase in PVN Gαi_2_ protein expression in multiple salt-resistant rat phenotypes in response to elevated sodium intake, and the absence of this response in the DSS rat, is of high physiological significance because of the pivotal role that the PVN plays in the neural network that influences sympathetic outflow and blood pressure regulation. Debate exists regarding the underlying cause of salt-sensitive hypertension, which could result from either over-activity of the sympathetic nervous system (Osborn et al., 2009) or excessive salt reabsorption at the level of the kidneys (Montani and Van Vliet, 2009). Our studies, as well as those of Mu et al. (2011); Mu et al., suggest that the underlying pathogenesis of salt-sensitive hypertension involves integration of both the central nervous system and the kidneys and we demonstrate that this communication is regulated by the renal sympathetic nerves via a CNS Gαi_2_ protein pathway. The potential translational impact of our findings of a role of Gαi_2_ proteins in blood pressure regulation is suggested by reports of single nucleotide polymorphisms (SNPs) in the human GNAI2 gene. SNPs in the GNAI2 gene are associated with increased hypertension risk in Caucasian Italians and in the Millennium Genome Project for Hypertension in Japan (Menzaghi et al., 2006; Kohara et al., 2008), providing the first evidence of the potential utility of GNAI2 SNPs as a biomarker of hypertension risk. However, at present the association between human GNAI2 SNPs and the salt-sensitivity of blood pressure remains to be investigated. Our findings of a direct role of the renal sympathetic nerves in the pathophysiology of salt-sensitive hypertension provides renewed support for the decades of evidence that have documented a role of the renal sympathetic nerves in the pathogenesis of hypertension in multiple animal models (DiBona and Esler, 2010) and in human hypertension (Schlaich et al., 2009). The potential clinical applicability of these data is provided by the recent renal denervation studies in humans that have resulted in a persistent reduction in blood pressure (Esler et al., 2012; Krum et al., 2014). We acknowledge that the clinical efficacy of renal nerve ablation in resistant hypertensive patients remains to be definitively established following recent studies, which failed to report reduced blood pressure in resistant hypertensive patients (Bhatt et al., 2014; Bakris et al., 2015). However, based upon the successful renal ablation trials, it has been speculated that the success of this clinical procedure reflects, in part, reduced hypothalamic-mediated sympathetic outflow (DiBona and Esler, 2010)—a hypothesis congruent with our current data. Collectively, our data suggest a novel molecular/cellular target (brain Gαi_2_ proteins) at which new therapies can be directed to alter systemic cardiovascular parameters (e.g., antihypertensive medications) and/or renal excretory function (natriuretic compounds) to treat the multiple disease states that feature sympathoexcitation and the impaired renal handling of sodium, such as salt-sensitive hypertension or congestive heart failure.

## Conflict of interest statement

The authors declare that the research was conducted in the absence of any commercial or financial relationships that could be construed as a potential conflict of interest.

## Abbreviations

AVP: vasopressin
CNS: central nervous system
DSR: Dahl salt-resistant
DSS: Dahl salt-sensitive
GPCR: G-protein coupled receptor
i.c.v.: intracerebroventricular
PVN: paraventricular nucleus
M: molar
NE: norepinephrine
N/OFQ: nociceptin/orphanin FQ
NOP: nociceptin/orphanin FQ receptor
SD: Sprague–Dawley
VE: volume expansion.

## References

[B1] Akine A. Montanaro M. Allen A. M. (2003). Hypothalamic paraventricular nucleus inhibition decreases renal sympathetic nerve activity in hypertensive and normotensive rats. Auton. Neurosci. 108, 17–21. 10.1016/j.autneu.2003.08.009 14614960

[B2] Bakris G. L. Townsend R. R. Flack J. M. Brar S. Cohen S. A. D'Agostino R.. (2015). 12-month blood pressure results of catheter-based renal artery denervation for resistant hypertension: the SYMPLICITY HTN-3 trial. J. Am. Coll. Cardiol. 65, 1314–1321. 10.1016/j.jacc.2015.01.037 25835443

[B3] Bayorh M. A. Ogbolu E. C. Williams E. Thierry-Palmer M. Sanford G. Emmett N.. (1998). Possible mechanisms of salt-induced hypertension in Dahl salt-sensitive rats. Physiol. Behav. 65, 563–568. 10.1016/s0031-9384(98)00194-2 9877424

[B4] Bhatt D. L. Kandzari D. E. O'Neill W. W. D'Agostino R. Flack J. M. Katzen B. T. (2014). A controlled trial of renal denervation for resistant hypertension. *N. Engl. J*. Med. 370, 1393–1401. 10.1056/NEJMoa1402670 24678939

[B5] Bie P. (2009). Blood volume, blood pressure and total body sodium: internal signalling and output control. Acta. Physiol. 195, 187–196. 10.1111/j.1748-1716.2008.01932.x 18983442

[B6] Brooks D. P. Share L. Crofton J. T. (1986). Central adrenergic control of vasopressin release. Neuroendocrinology 42, 416–420. 10.1159/000124480 3703161

[B7] Brooks V. L. Haywood J. R. Johnson A. K. (2005). Translation of salt retention to central activation of the sympathetic nervous system in hypertension. Clin. Exp. Pharmacol. Physiol. 32, 426–432. 10.1111/j.1440-1681.2005.04206.x 15854153

[B8] Cabral A. D. Kapusta D. R. Kenigs V. A. Varner K. J. (1998). Central alpha2-receptor mechanisms contribute to enhanced renal responses during ketamine-xylazine anesthesia. Am. J. Physiol. 275, R1867–R1874. 9843875 10.1152/ajpregu.1998.275.6.R1867

[B9] Calo G. Guerrini R. Rizzi A. Salvadori S. Burmeister M. Kapusta D. R.. (2005). UFP-101, a peptide antagonist selective for the nociceptin/orphanin FQ receptor. CNS Drug. Rev. 11, 97–112. 10.1111/j.1527-3458.2005.tb00264.x 16007234 PMC6741746

[B10] Carmichael C. Y. Wainford R. D. (2015). Hypothalamic signaling mechanisms in hypertension. Curr. Hypertens. Rep. 17, 39. 10.1007/s11906-015-0550-4 25860531 PMC4392165

[B11] Center for Disease Control Prevention (CDC) (2010). Sodium intake among adults – United States, 2005–2006. Morb. Mortal. Wkly. Rep. 59, 746–749. 20577156

[B12] Chan J. S. C. Yung L. Y. Lee J. W. M. Wi Y. L. Pei G. Wong Y. H. (1998). Pertussis toxin-insensitive signaling of the ORL1 receptor: coupling to Gz and G16 proteins. J. Neurochem. 71, 2203–2210. 10.1046/j.1471-4159.1998.71052203.x 9798948

[B13] Coote J. H. (1995). Cardiovascular functions of the paraventricular nucleus of the hypothalamus. Biol. Signals 4, 142–149. 10.1159/000109434 8750940

[B14] Degoute C. S. (2007). Controlled hypotension: a guide to drug choice. Drugs 67, 1053–1076. 10.2165/00003495-200767070-00007 17488147

[B15] DiBona G. F. (2004). The sympathetic nervous system and hypertension. Hypertension 43, 147–150. 10.1161/01.HYP.0000113047.47711.fa 14707153

[B16] DiBona G. F. (2005). Neural control of the kidney. Am. J. Physiol. 289, R633–R641. 10.1152/ajpregu.00258.2005 16105818

[B17] DiBona G. F. Esler M. (2010). Translational Physiology: the antihypertensive effect of renal denervation. Am. J. Physiol. 298, R245–R253. 10.1152/ajpregu.00647.2009 19955493

[B18] DiBona G. F. Kopp U. C. (1997). Neural control of renal function. Physiol. Rev. 77, 75–197. 9016301 10.1152/physrev.1997.77.1.75

[B19] Eason M. G. Liggett S. B. (1995). Identification of a Gs coupling domain in the amino terminus of the third intracellular loop of the α2A-adrenergic receptor. Evidence for distinct structural determinants that confer Gs versus Gi coupling. J. Biol. Chem. 270, 24753–24760. 10.1074/jbc.270.42.24753 7559592

[B20] Ellison D. H. Brooks V. L. (2011). Renal nerves, WNK4, glucocorticoids, and salt transport. Cell Metab. 13, 619–620. 10.1016/j.cmet.2011.05.007 21641543 PMC3112177

[B21] Esler M. D. Krum H. Schlaich M. Schmieder R. E. Böhm M. Sobotka P. A.. (2012). Renal sympathetic denervation for the treatment of drug-resistant hypertension: one-year results from the Symplicity HTN-2 randomized, controlled trial. Circulation 126, 2976–2982. 10.1161/CIRCULATIONAHA.112.130880 23248063

[B22] Foss J. D. Fink G. D. Osborn J. W. (2013). Reversal of genetic salt-sensitive hypertension by targeted sympathetic ablation. Hypertension 61, 806–811. 10.1161/HYPERTENSIONAHA.111.00474 23381790 PMC3658449

[B23] Franco V. Oparil S. (2006). Salt Sensitivity, a determinant of blood pressure, cardiovascular disease and survival. J. Med. Coll. Nutr. 25, 247S–255S. 10.1080/07315724.2006.10719574 16772636

[B24] Frithiof R. Ramchandra R. Hood S. May C. Rundgren M. (2009). Hypothalamic paraventricular nucleus mediates sodium induced changes in cardiovascular and renal function in conscious sheep. Am. J. Physiol. 297, R185–R193. 10.1152/ajpregu.00058.2008 19439617

[B25] Fujita M. Fujita T. (2013). The role of the CNS in salt-sensitive hypertension. Curr. Hypertens. Rep. 15, 390–394. 10.1007/s11906-013-0358-z 23689978

[B26] Gellai M. Edwards R. M. (1988). Mechanism of alpha 2-adrenoceptor agonist-induced diuresis. Am. J. Physiol. 255, F317–F323. 2900606 10.1152/ajprenal.1988.255.2.F317

[B27] Grindstaff R. R. Cunningham J. T. (2001). Cardiovascular regulation of vasopressin neurons in the supraoptic nucleus. Exp. Neurol. 171, 219–226. 10.1006/exnr.2001.7745 11573974

[B28] Grisk O. Dibona G. F. (1998). Influence of arterial baroreceptors and intracerebroventricular guanabenz on synchronized renal nerve activity. Acta. Physiol. Scand. 163, 209–218. 10.1046/j.1365-201x.1998.00357.x 9715732

[B29] Gullapalli S. Ramarao P. (2002). Role of L-type Ca2+ channels in pertussis toxin induced antagonism of U50, 488H analgesia and hypothermia. Brain. Res. 946, 191–191. 10.1016/S0006-8993(02)02880-9 12137921

[B30] Gunnet J. W. Wines P. Xiang M. Rybczynski P. Andrade-Gordon P. de Garavilla L.. (2008). Pharmacological characterization of RWJ-676070, a dual vasopressin V(1A)/V(2) receptor antagonist. Eur. J. Pharmacol. 590, 333–342. 10.1016/j.ejphar.2008.06.010 18599033

[B31] Guyton A. C. (1991). Blood-pressure control: special role of the kidneys and body fluids. Science 252, 1813–1116. 10.1126/science.2063193 2063193

[B32] Hadjimarkou M. M. Silva R. M. Rossi G. C. Pasternak G. W. Bodnar R. J. (2002). Feeding induced by food depravation is differentially reduced by G-protein α-subunit antisense probes in rats. Brain Res. 955, 45–54. 10.1016/S0006-8993(02)03361-9 12419520

[B33] Haselton J. R. Goering J. Patel K. P. (1994). Parvocellular neurons in the paraventricular nucleus are involved in the reduction in renal nerve discharge during isotonic volume expansion. J. Auton. Nerv. Syst. 50, 1–11. 10.1016/0165-1838(94)90117-1 7844308

[B34] Hawes B. E. Fried S. Yao X. Weig B. Graziano M. P. (1998). Nociceptin (ORL-1) and μ-opioid receptors mediate mitogen-activated protein kinase activation in CHO cells through a Gi-coupled signaling pathway: evidence for distinct mechanisms of agonist-mediated desensitization. J. Neurochem. 71, 1024–1033. 10.1046/j.1471-4159.1998.71031024.x 9721727

[B35] He F. J. Markandu N. D. Sagnella G. A. de Wardener H. E. MacGregor G. A. (2005). Plasma sodium: ignored and underestimated. Hypertension 45, 98–102. 10.1161/01.HYP.0000149431.79450.a2 15557392

[B36] Hein L. (2006). Adrenoceptors and signal transduction in neurons. Cell Tissue Res. 326, 541–551. 10.1007/s00441-006-0285-2 16896948

[B37] Huang B. S. Leenen F. H. (1998). Both brain angiotensin II and ‘ouabain’ contribute to sympathoexcitation and hypertension in Dahl S rats on high salt intake. Hypertension 32, 1028–1033. 10.1161/01.HYP.32.6.1028 9856968

[B38] Jeong S. W. Ikeda S. R. G. (1998). Protein α subunit Gαz couples neurotransmitter receptors to ion channels in sympathetic neurons. Neuron 21, 1201–1212. 10.1016/S0896-6273(00)80636-4 9856474

[B39] Kakiya S. Murase T. Aimra H. Yokoi H. Iwasaki Y. Miura Y.. (2000). Role of endogenous nociceptin in the regulation of arginine vasopressin release in conscious rats. Endocrinology 141, 4466–4471. 10.1210/endo.141.12.7809 11108256

[B40] Kandlikar S. S. Fink G. D. (2011). Splanchnic sympathetic nerves in the development of mild DOCA-salt hypertension. Am. J. Physiol. 301, H1965–H1973. 10.1152/ajpheart.00086.2011 PMC3213971 21890693

[B41] Kapusta D. R. Chang J. K. Kenigs V. A. (1999). Central administration of [Phe1Ψ (CH2-NH)Gly2]nociceptin(1–13)-NH2 and orphanin FQ/nociceptin (OFQ/N) produce similar cardiovascular and renal responses in conscious rats. *J. Pharmacol. Exp*. Ther. 289, 173–180. 10087001

[B42] Kapusta D. R. Kenigs V. A. (1999). Cardiovascular and renal responses produced by central orphanin FQ/nociceptin occur independent of renal nerves. Am. J. Physiol. 77, R987–R995. 10516236 10.1152/ajpregu.1999.277.4.R987

[B43] Kapusta D. R. Obih J. C. (1995). Central kappa opioids blunt the renal excretory responses to volume expansion by a renal nerve-dependent mechanism. J. Pharmacol. Exp. Ther. 273, 199–205 7714767

[B44] Kapusta D. R. Pascale C. L. Kuwabara J. T. Wainford R. D. (2013). CNS Gαi_2_–subunit proteins maintain salt-resistance via a renal nerve dependent sympathoinhibitory pathway. Hypertension 61, 368–375. 10.1161/HYPERTENSIONAHA.111.00014 23213191 PMC3562703

[B45] Kapusta D. R. Pascale C. L. Wainford R. D. (2012). Brain heterotrimeric Gαi_2_-subunit protein-gated pathways mediate central sympathoinhibition to maintain fluid and electrolyte homeostasis during stress. FASEB J. 26, 2776–2787. 10.1096/fj.11-196550 22459149 PMC3382099

[B46] Kapusta D. R. Sezen S. F. Chang J. K. Lippton H. Kenigs V. A. (1997). Diuretic and antinaturetic responses produced by the endogenous opioid-like peptide, nociceptin (orphanin FQ). Life Sci. 60, PL15–PL21. 8995537 10.1016/s0024-3205(96)00593-0

[B47] Katada T. Ui M. (1982). Direct modification of the membrane adenylate cyclase system by islet-activating protein due to ADP-ribosylation of a membrane protein. *Proc. Natl. Acad. Sci*. U.S.A. 79, 3129–3133. 10.1073/pnas.79.10.3129 PMC346367 6954463

[B48] Kenney M. J. Weiss M. L. Haywood J. R. (2003). The paraventricular nucleus: an important component of the central neurocircuitry regulating sympathetic nerve outflow. Acta. Physiol. Scand. 177, 7–15. 10.1046/j.1365-201x.2003.01042.x 12492774

[B49] Kohara K. Tabara Y. Nakura J. Imai Y. Ohkubo T. Hata A.. (2008). Identification of hypertension-susceptibility genes and pathways by a systemic multiple candidate gene approach: the millennium genome project for hypertension. Hypertens. Res. 31, 203–212. 10.1291/hypres.31.203 18360038

[B50] Kompanowska-Jezierska E. Wolff H. Kuczeriszka M. Gramsbergen J. B. Walkowska A. Johns E. J. (2008). Renal nerves and nNOS: roles in natriuresis of acute isovolumetric sodium loading in conscious rats. Am. J. Physiol. 294, R1130–R1139. 10.1152/ajpregu.00908.2007 18234741

[B51] Kondo K. Murase T. Otake K. Ito M. Kurimoto F. Oiso Y. (1993). Galanin as a physiological neurotransmitter in hemodynamic control of arginine vasopressin release in rats. Neuroendocrinology 57, 224–229. 10.1159/000126363 7685503

[B52] Kotchen T. A. Cowley A. W. Jr Frohlich E. D. (2013). Salt in health and disease—a delicate balance. N. Engl. J. Med. 368, 1229–1237. 10.1056/nejmra1212606 23534562

[B53] Krowicki Z. K. Kapusta D. R. (2006). Tonic nociceptinergic inputs to neurons in the hypothalamic paraventricular nucleus contribute to sympathetic vasomotor tone and water and electrolyte homeostasis in conscious rats. J. Pharmacol. Exp. Ther. 317, 446–453. 10.1124/jpet.105.094441 16407463

[B54] Krum H. Schlaich M. P. Sobotka P. A. Bohm M. Mahfoud F. Rocha-Singh K. (2014). Percutaneous renal denervation in patients with treatment-resistant hypertension: final 3-year report of the Symplicity HTN-1 study. Lancet 282, 622–629. 10.1016/S0140-6736(13)62192-3 24210779

[B55] Landgraf R. Malkinson T. Horn T. Veale W. L. Lederis K. Pittman Q. J. (1990). Release of vasopressin and oxytocin by paraventricular stimulation in rats. Am. J. Physiol. 258, R155–R159. 2301628 10.1152/ajpregu.1990.258.1.R155

[B56] Lastra G. Dhuper S. Johnson M. S. Sowers J. R. (2010). Salt, aldosterone, and insulin resistance: impact on the cardiovascular system. Nat. Rev. Cardiol. 7, 577–584. 10.1038/nrcardio.2010.123 20697411

[B57] Leenen F. H. Ruzicka M. Huang B. S. (2002). The brain and salt sensitive hypertension. Curr. Hypertens. Rep. 4, 129–135. 10.1007/s11906-002-0037-y 11884268

[B58] Lohmeier T. E. Hildebrandt W. Hood W. A. (1999). Renal nerves promote sodium excretion during long-term increases in salt intake. Hypertension 33, 487–492. 10.1161/01.HYP.33.1.487 9931153

[B59] Manunta P. Hamlyn J. M. Simonini M. Messaggio E. Lanzani C. Bracale M.. (2011). Endogenous ouabain and the renin-angiotensin-aldosterone system: distinct effects on Na handling and blood pressure in human hypertension. J. Hypertens. 29, 349–356. 10.1097/hjh.0b013e32833ea821 20842047 PMC3521520

[B60] Mattson D. L. Dwinell M. R. Greene A. S. Kwitek A. E. Roman R. J. Jacob H. J. (2008). Chromosome substitution revels the genetic basis of Dahl salt-sensitive hypertension and renal disease. *Am. J. Physiol*. Renal Physiol. 295, F837–F842. 10.1152/ajprenal.90341.2008 PMC2536867 18653478

[B61] May C. N. Frithiof R. Hood S. G. McAllen R. M. McKinley M. J. Ramchandra R. (2009). Specific control of sympathetic nerve activity to the mammalian heart and kidney. Exp. Physiol. 95, 34–40. 10.1113/expphysiol.2008.046342 19617268

[B62] McLoone V. I. Ringwood J. V. Van Vliet B. N. (2009). A multi-component model of the dynamics of salt-induced hypertension in Dahl-S rats. BMC Physiol. 9:20. 10.1186/1472-6793-9-20 19874603 PMC2785758

[B63] Menegaz R. G. Kapusta D. R. Mauad H. de Melo Cabral A. (2001). Activation of alpha(2)-receptors in the rostral ventrolateral medulla evokes natriuresis by a renal nerve mechanism. Am. J. Physiol. 281, R98–R107. 10.1152/ajpregu.2001.281.1.R98 11404283

[B64] Meneton P. Jeunemaitre X. de Wardener H. E. MacGregor G. A. (2005). Links between dietary salt intake, renal salt handling, blood pressure, and cardiovascular disease. Physiol. Rev. 85, 679–715. 10.1152/physrev.00056.2003 15788708

[B65] Menzaghi C. Paroni G. De Bonis C. Soccio T. Marucci A. Bacci S.. (2006). The-318 C>G single-nucleotide polymorphism in GNAI2 gene promoter region impairs transcriptional activity through specific binding of Sp1 transcription factor and is associated with high blood pressure in Caucasians from Italy. J. Am. Soc. Nephrol. 17, S115–S119. 10.1681/asn.2005121340 16565233

[B66] Montani J. P. Van Vliet B. N. (2009). Understanding the contribution of Guyton's large circulatory model to long-term control of arterial pressure. Exp. Physiol. 94, 382–388. 10.1113/expphysiol.2008.043299 19286638

[B67] Mu S. Shimosawa T. Ogura S. Wang H. Uetake Y. Kawakami-Mori F.. (2011). Epigenetic modulation of the renal β-adrenergic-WNK4 pathway in salt-sensitive hypertension. Nat. Med. 17, 573–580. 10.1038/nm0811-1020 21499270

[B68] Nasman J. Kukkonen J. P. Ammoun S. Akerman K. E. (2001). Role of G protein availability in differential signaling by alpha 2-adrenoceptors. Biochem. Pharmacol. 62, 913–922. 10.1016/s0006-2952(01)00730-4 11543726

[B69] Nielsen S. DiGiovanni S. R. Christensen E. I. Knepper M. A. Harris H. W. (1993). Cellular and subcellular immunolocalization of vasopressin-regulated water channel in rat kidney. *Proc. Natl. Acad. Sci*. U.S.A. 90, 11663–11667. 10.1073/pnas.90.24.11663 PMC48044 8265605

[B70] Osborn J. W. Averina V. A. Fink G. D. (2009). Current computational models do not reveal the importance of the nervous system in long-term control of arterial pressure. Exp. Physiol. 94, 389–396. 10.1113/expphysiol.2008.043281 19286640 PMC2684060

[B71] Osborn J. W. Collister J. P. Guzman P. (2008). Effect of peripheral sympathetic nerve dysfunction on salt sensitivity of arterial pressure. Clin. Exp. Pharmacol. Physiol. 35, 273–279. 10.1111/j.1440-1681.2007.04827.x 17973927

[B72] Patel C. B. Noor N. Rockman H. A. (2010). Functional selectivity in adrenergic and angiotensin signalling systems. Mol. Pharmacol. 78, 983–992. 10.1124/mol.110.067066 20855464 PMC2993470

[B73] Patel K. P. (1991). Central alpha-2 adrenergic mechanisms in the renal nerve mediated natriuresis and diuresis produced by acute volume expansion. J. Auton. Nerv. Syst. 36, 47–54. 10.1016/0165-1838(91)90129-q 1661306

[B74] Reinscheid R. K. Nothacker H. P. Bourson A. Ardati A. Henningsen R. A. Bunzow J. R.. (1995). Orphanin FQ: a neuropeptide that activates an opioidlike G protein-coupled receptor. Science 270, 792–794. 10.1126/science.270.5237.792 7481766

[B75] Remaury A. Larrouy D. Daviaud D. Rouot B. Paris H. (1993). Coupling of the alpha 2-adrenergic receptor to the inhibitory G-protein Gi and adenylate cyclase in HT29 cells. Biochem. J. 292, 283–288. 8099279 10.1042/bj2920283 PMC1134302

[B76] Rodriguez-Iturbe B. Vaziri N. D. (2007). Salt-sensitive hypertension: update on novel findings. Nephrol. Dial. Transplant. 22, 992–995. 10.1093/ndt/gfl757 17210585

[B77] Rossi G. C. Standifer K. M. Pasternak G. W. (1995). Differential blockade of morphine, and morphine-6 beta-glucuronide analgesia by antisense oligodeoxynucleotides directed against MOR-1 and G-protein alpha subunits in rats. Neurosci. Lett. 198, 99–102. 10.1016/0304-3940(95)11977-5 8592651

[B78] Ruffolo R. R. Jr. Nichols A. J. Stadel J. M. Hieble J. P. (1991). Structure and function of alpha-adrenoceptors. Pharmacol. Rev. 43, 475–505 1685567

[B79] Ruginsk S. G. Oliveira F. R. Margatho L. O. Vivas L. Elias L. L. Antunes-Rodrigues J. (2007). Glucocorticoid modulation of neuronal activity and hormone secretion induced by blood volume expansion. Exp. Neurol. 206, 192–200. 10.1016/j.expneurol.2007.04.012 17553493

[B80] Schlaich M. Sobotka P. A. Krum H. Lambert E. Esler M. D. (2009). Renal sympathetic ablation for the treatment of uncontrolled hypertension. New Engl. J. Med. 361, 932–934. 10.1056/NEJMc0904179 19710497

[B81] Serino R. Ueta Y. Hanamiya M. Nomura M. Yamamoto Y. Yamaguchi K. I.. (2001). Increased levels of hypothalamic neuronal nitric oxide synthase and vasopressin in salt-loaded Dahl rat. Auton. Neurosci. 87, 225–235. 10.1016/S1566-0702(00)00279-4 11476283

[B82] Silva R. M. Rossi G. C. Mathis J. P. Standifer K. M. Pasternak G. W. Bodnar R. J. (2000). Morphine and morphinw-6-beta-glucaronide-induced feeding are differentially reduced by G-protein alpha-subunit antisense in rats. Brain Res. 876, 62–75. 10.1016/S0006-8993(00)02621-4 10973594

[B83] Singer D. R. Markandu N. D. Buckley M. G. Miller M. A. Sagnella G. A. MacGregor G. A. (1998). Contrasting endocrine responses to acute oral compared with intravenous sodium loading in normal humans. Am. J. Physiol. 244, F111–F119. 9458830 10.1152/ajprenal.1998.274.1.F111

[B84] Standifer K. M. Rossi G. C. Pasternak G. W. (1996). Differential blockade of opioid analgesia by antisense oligodeoxynucleotides directed against various G protein alpha subunits. Mol. Pharmacol. 50, 293–298. 8700136

[B85] Stocker S. D. Monahan K. D. Browning K. N. (2013). Neurogenic and sympathoexcitatory actions of NaCl in Hypertension. Curr. Hypertens. Rep. 5, 538–546. 10.1007/s11906-013-0385-9 24052211 PMC4017866

[B86] Toney G. M. Chen Q. H. Cato M. J. Stocker S. D. (2003). Central osmotic regulation of sympathetic nerve activity. Acta. Physiol. Scand. 177, 43–55. 10.1046/j.1365-201X.2003.01046.x 12492778

[B87] Tso W. Wong Y. H. (2006). Opioid receptor-like (ORL1) receptor utilizes both GoA and GoB for signal transduction. Prot. Pep. Lett. 13, 437–441. 10.2174/092986606776819547 16800795

[B88] Van Vliet B. N. Chafe L. L. Halfyard S. J. Leonard A. M. (2006). Distinct rapid and slow phases of salt-induced hypertension in Dahl salt-sensitive rats. J. Hypertens. 24, 1599–1606. 10.1097/01.hjh.0000239296.25260.e0 16877963

[B89] Wainford R. D. Carmichael C. Y. Pascale C. L. Kuwabara J. T. (2015). Gαi_2_-protein mediated signal transduction: a CNS molecular mechanism countering the development of sodium-dependent hypertension. Hypertension 65, 178–186. 10.1161/HYPERTENSIONAHA.114.04463 25312437 PMC4268057

[B90] Wainford R. D. Kapusta D. R. (2009). Chronic high-NaCl intake prolongs the cardiorenal responses to central N/OFQ and produces regional changes in the endogenous brain NOP receptor system. Am. J. Physiol. 296, R280–R288. 10.1152/ajpregu.00096.2008 PMC2643980 18987291

[B91] Wainford R. D. Kapusta D. R. (2010). Hypothalamic paraventricular nucleus G alpha q subunit protein pathways mediate vasopressin dysregulation and fluid retention in salt-sensitive rats. Endocrinology 151, 5403–5414. 10.1210/en.2010-0345 20861238 PMC2954710

[B92] Wainford R. D. Kapusta D. R. (2012). Functional selectivity of central Gα-subunit proteins in mediating the cardiovascular and renal excretory responses evoked by central α(2)-adrenoceptor activation *in vivo*. Br. J. Pharmacol. 166, 210–220. 10.1111/j.1476-5381.2011.01662.x 21895632 PMC3415649

[B93] Wainford R. D. Kurtz K. Kapusta D. R. (2008). Central G-alpha subunit protein-mediated control of cardiovascular function, urine output, and vasopressin secretion in conscious Sprague-Dawley rats. Am. J. Physiol. 295, R535–R542. 10.1152/ajpregu.00043.2008 PMC2519935 18525017

[B94] Wainford R. D. Pascale C. L. Kuwabara J. T. (2013). Brain Gαi_2_–subunit protein-gated pathways are required to mediate the centrally evoked sympathoinhibitory mechanisms activated to maintain sodium homeostasis. J. Hypertens. 31, 747–757. 10.1097/HJH.0b013e32835ebd54 23391983

[B95] Whelton P. K. Appel L. J. Sacco R. L. Anderson C. A. Antman E. M. Campbell N.. (2012). Sodium, blood pressure, and cardiovascular disease: further evidence supporting the American Heart Association sodium reduction recommendations. Circulation 126, 2880–2889. 10.1161/CIR.0b013e318279acbf 23124030

[B96] Yatabe M. S. Yatabe J. Yoneda M. Watanabe T. Otsuki M. Felder R. A.. (2010). Salt sensitivity is associated with insulin release, sympathetic overactivity, and decreased suppression of circulating renin activity in lean patients with essential hypertension. Am. J. Clin. Nutr. 91, 77–82. 10.3945/ajcn.2009.29028 20444953

[B97] Yung L. Y. Joshi S. A. Chan R. Y. Chan J. S. Pei G. Wong Y. H. (1999). G alphaL1 (Galpha14) couples the opioid receptor-like 1 receptor to stimulation of phospholipase C. J. Pharma. Exp. Ther. 288, 232–238. 9862775

